# 2050: Improving evidence synthesis: Partnering with the Center for Clinical & Translational Science to build a Systematic Review Core

**DOI:** 10.1017/cts.2017.155

**Published:** 2018-05-10

**Authors:** Melissa L. Rethlefsen, Mellanye Lackey, Michelle Fiander, Mary McFarland

**Affiliations:** The University of Utah School of Medicine, Salt Lake City, UT, USA

## Abstract

OBJECTIVES/SPECIFIC AIMS: To improve the quality of evidence synthesis projects, including systematic reviews and other comparative effectiveness reviews, at the University of Utah. METHODS/STUDY POPULATION: Systematic reviews and other types of evidence syntheses are best when collaborative teams with expertise in multiple disciplines participate, including content experts, librarians and information specialists, systematic review methodologists, and statisticians. The Center for Clinical & Translational Science (CCTS), due to its interdisciplinary nature, connectivity to clinical experts, and existing Cores of methodologists, presented an opportune location for a Systematic Review Core. We designed the Systematic Review Core to focus on 2 primary aspects of evidence synthesis support: overall systematic review methodology guidance and in-depth information retrieval planning and execution. After establishing a conceptual partnership, a new position, Evidence Retrieval and Synthesis Librarian, was created to build capacity within the Core. RESULTS/ANTICIPATED RESULTS: Close connections with the CCTS’s Population Health Research Foundation have led to better interdisciplinary coverage of systematic reviews and other evidence syntheses produced by the University of Utah. We are able to partner with statisticians and clinical experts from formulating the question to completing the final manuscript. Hourly rates charged through a cost recovery model have enabled us to grow our staff able to work on the Core, as well as offset costs for major databases and resources these bibliographic data-heavy research methods require. After 1 year of existence, the Core is already at maximum capacity, with no sign of slowing. Projects have ranged from brief consultations to highly intense interactions for the duration of the research spectrum. We have also been added as key personnel to grants with systematic review components. DISCUSSION/SIGNIFICANCE OF IMPACT: Systematic reviews and other evidence syntheses are a labor-intense, interdisciplinary team effort that fit well within the scope of CTSA’s. They are a key component of the translation of science to practice, and can be used at all stages of the translational science spectrum. Quality of systematic reviews remains poor, particularly surrounding protocol development, sensitive search strategy design and reporting, and overall reporting. Librarians and information specialist involvement has been shown to positively correlate to the search strategy design and reporting aspects of systematic reviews, and librarians and information specialists increasingly act as systematic review methodologists. By including librarians and information specialists as part of the CTSA’s official Core structure, these systematic review methodologists are able to connect with statisticians, other methodologists, and clinical experts in a nexus of interdisciplinarity. At the University of Utah, the visibility and structure provided by the CCTS helps the Systematic Review Core with promotion, creating connections and opportunities for collaboration across the campus. This partnership has already led to increased uptake in services, and over time, we believe it will increase the quality of the science produced. CTSA’s have a natural partner with their health science library colleagues in translational science, as shown by this model.

